# Elevated Liver Enzymes in an Adult Male With Anorexia Nervosa: A Case Report

**DOI:** 10.7759/cureus.31367

**Published:** 2022-11-11

**Authors:** Nikolaos Kintrilis

**Affiliations:** 1 Emergency Department, 401 General Military Hospital of Athens, Athens, GRC

**Keywords:** hypoglycemia, refeeding, hepatic autophagy, hypoperfusion, transaminitis, liver function test (lft), malnourishment, anorexia nervosa (an)

## Abstract

Anorexia nervosa is a prevalent eating disorder often accompanied by various medical complications, along with increased liver serum enzymes, especially transaminases. Here, we describe the case of an adult male patient admitted to a general hospital. The patient presented with malnourishment arising from AN and severely affected liver function tests.

## Introduction

Anorexia nervosa (AN) is one of the top three most prevalent eating disorders, along with binge eating disorder and bulimia nervosa, with a lifetime prevalence of 0.16% according to a recent systematic review and meta-analysis [1]. Criteria to diagnose the disorder based on the Diagnostic and Statistical Manual of Mental Disorders, fourth edition (DSM-IV) include weight lower than 85% of normal weight/body mass index (BMI) ≤17.5 kg/m^2^ and amenorrhea [2], while DSM-V requires weight lower than the lowest value of normal weight or lower than the lowest predicted value of children and juveniles and a course of at least three months [3]. Individuals suffering from AN present disturbed eating behavior along with an overwhelming desire to lose or continue losing weight and maintain a slender physique, as well as disturbed body image. The disease most commonly manifests as restriction of caloric intake and can be accompanied by binge eating along with purging or excessive energy expenditure, all of which lead to continued weight loss [1]. Although the disease can occur in both sexes at any age, it shows a predilection for adolescent females, with the prevalence of AN in females up to 10 times higher than that in males [4]. The overall incidence of the disease has been somewhat stable over the last decades, although the incidence among younger individuals has increased owing possibly both to an earlier onset of adulthood and earlier detection due to better screening methods [1].

The disorder often goes hand-in-hand with various chronic illnesses and comorbidities, along both the physical and mental spectra, as well as high mortality rates, representing a serious mental disorder that needs a multifactorial approach and often presents as a challenge to psychiatrists and physicians involved in its treatment. Common sequelae of the disease include hematologic and bone marrow abnormalities, disordered electrolytes, cardiac complications, low bone mass/osteoporosis, and fractures, as well as hormonal changes and amenorrhea. Most common clinical insults from the gastrointestinal tract include constipation, pancreatitis, reduced appetite, and early satiety, as well as pathological increases in liver enzymes, especially serum aspartate aminotransferase (AST) and serum alanine aminotransferase (ALT), depicting an injury to liver cells [5]. Although the exact etiopathogenetic mechanism involved in the clinical insult is not fully explained, it is believed that the process is mediated through the phenomenon of liver autophagy occurring during starvation, wherein liver cells inevitably break down and consume themselves when all other nutrients have been exhausted in the process of starving oneself [6]. A second proposed etiological mechanism involves ischemic hepatitis occurring from marked hypotension and hypoglycemia in AN patients, conditions that lead to organ hypoperfusion, a theory supported by rapid decreases in affected biochemical values when rehydration and gradual refeeding are ensured, leading to the enhancement of vital values and liver reperfusion [7].

Here, we examine the case of an adult male patient admitted to a tertiary hospital with malnourishment and severe hepatic biochemistry abnormalities due to AN.

## Case presentation

Case history and examination

A 30-year-old male without any previous medical or surgical history, but with a history of anxiety and disordered eating, presented to the Emergency Department of the hospital. Upon arrival for evaluation, the patient collapsed and almost lost consciousness and was admitted for further evaluation. The patient received no medications at the time. He mentioned no alcohol, substance, or tobacco use whatsoever. His family history included treated breast cancer in his mother and no other physical or psychiatric conditions. Upon initial recovery and vital sign examination, the Glasgow Coma Scale score was 15/15. He was bradycardic with a heart rate (HR) of 33 beats per minute and hypotensive with a blood pressure (BP) of 87/49 mmHg. His respiratory rate (RR) was normal at 14 breaths per minute, and his temperature was unremarkable at 35.9°C. Capillary blood glucose level was 71 mg/dL. He appeared icteric, pale, and extremely weak, presenting with a mild tremor. At the time of presentation, his weight was 49.2 kg, and his height was 184 cm, with a BMI of 14.5 kg/m^2^. Clinical examination revealed cachexia and total body paleness. His torso was hairless while his extremities had lanugo hair. His skin and tongue showed signs of extreme dryness and were scaly. The rest of the clinical examination was unremarkable.

Blood was drawn for a complete blood count and full biochemical profile, the results of which are presented in Table 1. An electrocardiogram showed sinus bradycardia at 35 beats per minute without further abnormalities, and a chest X-ray was unremarkable. A cardiologic consult was requested to assess for possible myocardial involvement, with an echocardiogram being performed, which showed a normal heart size without any notable changes except for the remains of a Eustachian tube.

**Table 1:** Laboratory parameters upon arrival at the Emergency Department. WBC: white blood cells; Hgb: hemoglobin; Hct: hematocrit; RBC: red blood cells; AST: aspartate aminotransferase; ALT: alanine aminotransferase; LDH: lactate dehydrogenase; γGT: gamma-glutamyltransferase; ALKP: alkaline phosphatase; INR: international normalized ratio

Parameter	Result	Normal range
WBC (K/μL)	4.1	4.0–10.8
Hgb (g/dL)	11.1	13.5–17.9
Hct (%)	32.7	40.0–52.0
RBC (M/μL)	3.3	4.5–6.1
Platelets (K/μL)	77	150–440
AST (U/L)	839	5–40
ALT (U/L)	1,185	5–40
LDH (U/L)	484	200–460
γGT (U/L)	266	9–40
ALKP (U/L)	112	43–115
Glucose (mg/dL)	73	70–110
Urea (mg/dL)	78	10–50
Creatinine (mg/dL)	1.10	0.7–1.5
Total bilirubin (mg/dL)	0.70	0.20–1.20
Sodium (mmol/L)	138	135–150
Potassium (mmol/L)	4.2	3.6–5.0
INR	1.20	0.85–1.15

Case outcome and follow-up

**Figure 1:**
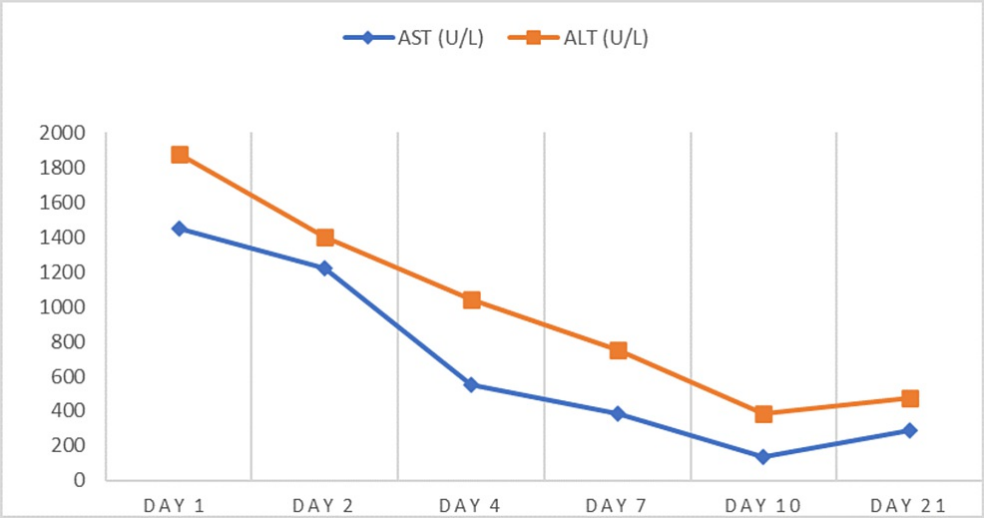
Trend of liver enzymes over the course of the patient’s hospitalization. AST: aspartate aminotransferase; ALT: alanine aminotransferase

The patient was admitted to the Internal Medicine Department of the hospital, where he was placed on intravenous dextrose solution and parenteral feeding for the first 24 hours, after which he refused further intravenous feeding. Evaluation for other potential sources of liver damage (hepatitis A, B, C viruses, hepatotropic viruses, human immunodeficiency virus, hemochromatosis, Wilson disease, and autoimmune hepatitis antibodies) was performed, with negative results. During the first 48 hours, biochemical parameters further worsened, and an upper abdominal ultrasound revealed no apparent disorders. After psychiatric and dietary evaluation, he was placed on a selective serotonin reuptake inhibitor (SSRI), and a consulting dietician suggested a closely supervised diet of 1,200 kcal by mouth, with a goal of gradually increasing energy intake every seven days. His weight further dropped to a minimum of 45.3 kg during his stay at the hospital for the lowest BMI of 13.4 kg/m^2^. He was discharged after 21 days at a weight of 51.9 kg with a discharge BMI of 15.3 kg/m^2^. His general condition and mood improved drastically over the course of his 21-day admission, with most biochemical tests normalizing or improving upon discharge with the exception of liver function tests. Table 2 depicts the evolution of the patient’s biochemical parameters over the course of his hospitalization, and Figure 1 shows the trend of his transaminases.

**Table 2:** Evolution of laboratory parameters over the patient’s hospitalization. WBC: white blood cells; Hgb: hemoglobin; Hct: hematocrit; RBC: red blood cells; AST: aspartate aminotransferase; ALT: alanine aminotransferase; LDH: lactate dehydrogenase; γGT: gamma-glutamyltransferase; ALKP: alkaline phosphatase; INR: international normalized ratio

Parameter	Day 1	Day 2	Day 4	Day 7	Day 10	Day 21	Normal range
WBC (K/μL)	4.0	4.0	3.7	3.7	3.0	4.1	4.0–10.8
Hgb (g/dL)	9.9	9.6	9.4	9.2	8.5	10.2	13.5–17.9
Hct (%)	28.5	27.3	28.3	27.1	24.2	30.0	40.0–52.0
RBC (M/μL)	3.0	2.9	2.8	2.7	2.5	3.0	4.5–6.1
Platelets (K/μL)	64	59	65	76	141	160	150–440
AST (U/L)	1,451	1,221	554	386	139	286	5–40
ALT (U/L)	1,875	1,404	1,043	750	388	477	5–40
LDH (U/L)	726	749	386	362	546	390	200–460
γGT (U/L)	278	289	242	246	214	200	9–40
ALKP (U/L)	128	145	125	120	102	108	43–115
Glucose (mg/dL)	93	89	80	87	90	79	70–110
Urea (mg/dL)	59	89	41	41	36	50	10–50
Creatinine (mg/dL)	0.80	0.80	1.00	0.70	0.90	0.90	0.7–1.5
Total bilirubin (mg/dL)	0.40	0.50	0.80	0.60	0.60	0.40	0.20–1.20
Sodium (mmol/L)	139	135	142	137	131	138	135–150
Potassium (mmol/L)	3.90	3.60	4.30	3.60	3.80	4.20	3.6–5.0
INR	1.22	1.18	1.12	1.17	1.18	1.10	0.85–1.15

The patient left the hospital on the 21st day after his admission on an SSRI and benzodiazepine to aid sleep, as well as an at-home diet plan to foster further weight gain over the following weeks, at least until a safe BMI of 17 kg/m^2^ is achieved. Psychotherapy twice a week on an outpatient basis was implemented, and the patient was invited for an outpatient clinical reassessment as well as a repetition of his liver function biochemical values once every week thereafter. Although his weight increase was not linear, possibly due to reported diet compliance issues after his hospital discharge, liver enzymes finally normalized approximately 10 weeks after his initial presentation.

## Discussion

AN represents a serious psychiatric disease manifesting as an eating disorder with a multitude of symptoms and clinical complications, with one of the most common being hepatic involvement. Liver damage may not be clinically apparent, presenting in the form of mild-to-severe liver enzyme derailment, especially elevations of AST and ALT. According to recent literature, these elevations, also mentioned under the term transaminitis, are more serious as the degree of being underweight worsens, and improve drastically when the refeeding process is initiated and the weight starts to restore [8]. There have been reports of patients with worsening hepatic function after initiation of parenteral nutrition, including the death of a patient from liver failure. Although it is yet unclear which patients will present with liver damage, risk factors associated with transaminitis have been described, namely, young age, low BMI, male sex, as well as being diagnosed with the pure restrictive variant of the disease [9]. Of note, many patients present with completely normal laboratory values, albeit being severely malnourished and underweight [8].

Even though the exact etiopathogenetic mechanisms remain unclear, two main processes have been proposed as mediators for the clinical phenomenon, namely, acute hypoperfusion and liver autophagy. During the acute phase of the disease, liver hypoperfusion owing to hypoglycemia and relative bradycardia inevitably leads to hypoperfusion and cell death, thus increasing liver enzymes [9]. Moreover, liver autophagy has been described as a mechanism for the organ to maintain blood glucose through gluconeogenesis, converting amino acids to glucose [10]. In the context of AN, a lack of building blocks and energy exhaustion at the later stages of the disease may initiate the process of liver autophagy, during which liver cells consume themselves and promote aminotransferase elevations [6].

## Conclusions

Here, we report the case of an adult male presenting with severe AST and ALT elevations due to AN malnourishment, without signs of steatosis or cholestasis and exclusion of other hepatic damage etiologies. During the initial hospitalization, enzymes continued to rise as the weight dropped a little more, insinuating that the more probable mechanism in our case was hypoperfusion and transient ischemic hepatitis. This case adds to the scarce literature in the field of adult male sufferers of AN. Further research to more precisely understand the underlying mechanisms of the disease is needed.
